# Rare Coronary Embolism Secondary to Cardioversion of Atrial Fibrillation

**DOI:** 10.7759/cureus.24354

**Published:** 2022-04-21

**Authors:** Deya Alkhatib, Basil Al-Sabeq

**Affiliations:** 1 Cardiology, The University of Tennessee Health Science Center, Memphis, USA; 2 Cardiology, Methodist University Hospital, Methodist Le Bonheur Healthcare/The University of Tennessee Health Science Center, Memphis, USA

**Keywords:** minoca, coronary embolism, microvascular obstruction (mvo), direct current cardioversion (dccv), atrial fibrillation (af), coronary artery embolism, cardiac magnetic resonance (cmr), myocardial infarction with non-obstructive coronary arteries (minoca)

## Abstract

The diagnosis and management of myocardial infarction with nonobstructive coronary arteries (MINOCA) are difficult due to its variable presentations, different causes, and challenging diagnostic approaches. Cardiac imaging modalities including cardiac magnetic resonance (CMR) are very useful tools for diagnosing and managing MINOCA. Myocardial infarction (MI) can be caused by coronary emboli that can be contributed to atrial fibrillation (AF). Rarely, coronary embolism with resultant MINOCA can occur after direct current cardioversion (DCCV) even in fully anticoagulated patients. We present a rare case of a coronary embolism following DCCV as well as a CMR finding of microvascular obstruction (MVO), which has not previously been reported after DCCV. This case also emphasizes the value of obtaining a CMR for patients with MINOCA.

## Introduction

Determining the precise etiology of myocardial infarction with nonobstructive coronary arteries (MINOCA) can be challenging. Cardiac magnetic resonance (CMR) imaging is very helpful in diagnosing, treating, and risk-stratifying patients with MINOCA. Cardiac emboli are one of the causes of myocardial infarction (MI), and atrial fibrillation (AF) accounts for approximately 15% of cardiac emboli [1]. It is exceedingly rare to have cardiac emboli after direct current cardioversion (DCCV) in adequately anticoagulated patients [2].

## Case presentation

**Figure 1:**
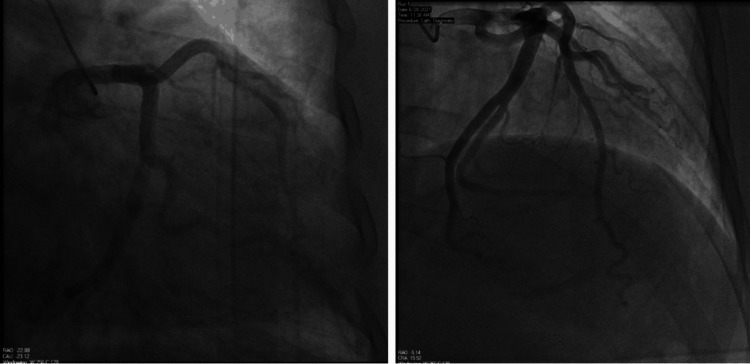
Coronary angiography demonstrated only minimal luminal coronary artery disease

A 51-year-old man with a past medical history of paroxysmal AF, hypertension, and morbid obesity presented to the emergency department with worsening dyspnea and lower extremity edema and was hospitalized for de novo acute decompensated heart failure (ADHF). He was noted to be in AF with a rapid ventricular response. Transthoracic echocardiography revealed severe systolic dysfunction with a left ventricular ejection fraction (LVEF) of 20-25%. Coronary angiography demonstrated only minimal luminal coronary artery disease (Figure 1). Differential diagnoses of this non-ischemic cardiomyopathy included tachycardiomyopathy. He underwent a transesophageal echocardiogram (TEE), which ruled out left atrial appendage (LAA) thrombus. Synchronized direct current cardioversion (DCCV) was successful in converting AF into normal sinus rhythm (NSR), but the patient reverted to AF within 24 hours. He was started on sotalol and apixaban and was cardioverted again while on antiarrhythmics after four weeks of anticoagulation, and NSR was sustained.

**Figure 2:**
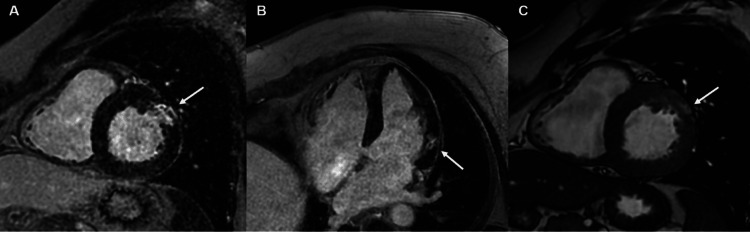
Cardiac magnetic resonance imaging Panel A: delayed gadolinium enhancement of the left ventricle in the short axis demonstrates a dense, transmural, focal area of hyperenhancement in the basal anterolateral segment with central hypoenhancement (arrow), consistent with myocardial infarct and microvascular obstruction. Panel B displays the infarct in a four-chamber view. Balanced steady-state free precession demonstrates hyperintense mid-myocardial signal relative to myocardium corresponding to the region of infarct, suggestive of lipomatous metaplasia (panel C, arrow)

Three months after the patient was diagnosed with heart failure with reduced ejection fraction (HFrEF), CMR was performed using a 1.5 T scanner (Signa Artist, GE Medical Systems, Milwaukee, WI). Retrospective ECG-gated breath-hold steady-state free precession cine images were obtained in short-axis slices at 10 mm intervals (slice thickness of 6 mm, 4 mm gap) and two-chamber, three-chamber, and four-chamber views. This revealed normal left ventricular size and ejection fraction and a hypokinetic basal anterolateral segment. Gadolinium-based contrast agent (MultiHance, Bracco Diagnostics, Princeton, NJ) was injected in a 15-mmol bolus and late gadolinium enhancement (LGE) images were acquired 10 minutes later by using an inversion recovery gradient echo sequence. This revealed dense transmural hyperenhancement in the basal anterolateral myocardium with central hypoenhancement consistent with microvascular obstruction (MVO, Figure 2). Moreover, the focal nature of hyperenhancement and the absence of wavefront phenomenon were more consistent with an embolic infarct. In hindsight, this patient met the criteria for MINOCA and was found to have evidence of MI secondary to coronary embolism, likely after DCCV (Table 1). In this case report, we highlight the role of CMR in MINOCA and the rare finding of MVO post-DCCV.

**Table 1:** Timeline of the patient's disease course

Time	Events
8 weeks prior to admission	Progressive dyspnea on exertion
Day 0	Admitted for acute decompensated heart failure and atrial fibrillation (AF) with a rapid ventricular response
Day 1	Transthoracic echocardiogram (TTE) showing left ventricular ejection fraction (LVEF) of 25-30% and regional wall motion abnormalities
Days 1-3	Heart rate control was achieved and volume status improved after intravenous diuresis
Day 4	Coronary angiography ruled out obstructive coronary artery disease. Therapeutic unfractionated heparin was switched to apixaban
Day 5	A transesophageal echocardiogram (TEE) ruled out an organized thrombus in the left atrial appendage
Day 5	Direct current cardioversion (DCCV) was successful in converting AF into normal sinus rhythm (NSR) with no complications. Started on sotalol for maintaining NSR
Day 6	Reverted to AF. Discharged home on sotalol and apixaban
Day 33	Returned for elective DCCV while therapeutically anticoagulated. Successful DCCV in converting AF into NSR. Discharged home in NSR
Day 90	Cardiac magnetic resonance (CMR) imaging showed LVEF recovery and normalization, and evidence of myocardial infarct and microvascular obstruction (MVO)

## Discussion

In order to diagnose MINOCA, three criteria must be met: diagnosis of acute MI, ruling out flow-limiting coronary artery disease using invasive angiography or coronary CT angiography (CCTA), and absence of obvious causes of acute MI. Differential diagnoses of MINOCA include plaque erosion or rupture with spontaneous recanalization, coronary embolism, spontaneous coronary artery dissection, microvascular disease, stress cardiomyopathy, and myocarditis. Due to the broad differential diagnosis of MINOCA, treatment and prognosis vary remarkably [3,4]. CMR is a powerful tool in assessing patients with MI due to its ability to diagnose acute and chronic MI, narrow the differential diagnosis, and determine the prognosis by assessing the scar burden. In patients with MINOCA, CMR should be obtained within seven days of presentation and preferably not in the first 24 hours to improve the diagnostic accuracy [3]. CMR protocol should include cine assessment of cardiac function and structure, T2 assessment of myocardial edema, and LGE to assess for macroscopic scar/fibrosis/infiltrative disease (both pattern and extent) [3]. LGE technique can be helpful in broadly categorizing MINOCA into ischemic versus non-ischemic hyperenhancement patterns. Ischemic MINOCA will have a subendocardial or transmural LGE pattern versus mid-myocardial or subepicardial in non-ischemic causes such as viral myocarditis [5]. Moreover, CMR can help categorize the type of ischemic LGE pattern based on appearance, as embolic infarcts often display a wedge-shaped hyperenhancement pattern and the absence of wavefront phenomenon with a predominance of scar in the subepicardium rather than subendocardium [6]. It is important to differentiate this atypical infarct pattern from common non-ischemic etiologies.

MVO is seen following coronary reperfusion therapy, especially after prolonged myocardial ischemia. It is caused by microvascular damage impairing blood flow through damaged capillaries and is the CMR visual representation of the no-reflow phenomenon [7,8]. On early or late gadolinium enhancement, MVO is seen as a dark central (hypoenhanced) focus within hyperenhanced myocardial scar, which is due to the inability of gadolinium to penetrate the damaged microvasculature. Correlated myocardial segments with MVO are more likely to have remodeling, wall-thinning, and poor functional recovery [9]. Identification of MVO can predict the risk of left ventricular remodeling and future risk of major adverse cardiac events; MVO on LGE is more prognostic than on early gadolinium enhancement [8,10]. MVO is typically seen after MI but may be iatrogenic such as after catheter ablation of ventricular tachycardia [8].

AF, infective endocarditis, and prosthetic valve thrombosis are the most common causes of coronary emboli; AF accounts for about 15% of coronary emboli [1]. Coronary embolism after DCCV of AF is exceedingly rare and seldom reported [2].

## Conclusions

This case illustrates the importance of obtaining CMR in patients with MINOCA. Although our patient did not present with symptoms of an MI, CMR was able to identify a silent embolic infarct after DCCV for AF. Despite its paucity, DCCV in AF patients can cause coronary embolism even in fully anticoagulated patients.
